# The Neural and Psychological Processes of Peer-Influenced Online Donation Decision: An Event-Related Potential Study

**DOI:** 10.3389/fpsyg.2022.899233

**Published:** 2022-05-20

**Authors:** Yuchen Ye, Pengtao Jiang, Wuke Zhang

**Affiliations:** ^1^Business School, Ningbo University, Ningbo, China; ^2^School of Information Science and Engineering, NingboTech University, Ningbo, China; ^3^Business School, University of Nottingham Ningbo China, Ningbo, China

**Keywords:** online donation, peer influence, number of donated peers, event-related potential, P2, N2, P3

## Abstract

With the rapid development of information and communication technology (ICT), social media-based donation platforms emerged.^1^ These platforms innovatively demonstrate peer information (e.g., number of donated peers) on the donation page, which inevitably brings the peer influence into donors’ donation decision process. However, how the peer influence will affect the psychological process of donation decisions are remained unknown. This study used the number of donated peers to examine the effects of peer influence on donors’ donation decisions and extracted event-related potential (ERP) from electroencephalographic data to explore the underlying psychological process. The behavioral results indicated that the number of donated peers positively influenced donors’ willingness to donate. The ERP results suggested that a larger number of donated peers might indicate a higher level of conformity and greater perceived emotional rewards, as a larger P2 amplitude was observed. Following the early processing of emotional stimuli, cognitive detection of decisional risk took place, and the donors reckoned a smaller number of donated peers as a high potential risk, which was reflected by a larger N2 amplitude. In the later stage, the larger number of donated peers, which represented a higher magnitude of prospective emotional rewards, led to a higher incentive to donate, and reflected in a larger amplitude of P3. Additionally, implications and future directions were discussed.

## Introduction

Charities have become an essential means of helping vulnerable and marginalized groups in society (Erceg et al., 2018). With the rapid development of social media and information and communication technology (ICT), healthcare information dissemination is even faster (Abbasi et al., 2018). It also facilitates the emergence and development of online donation platforms based on social media. Taking the latest authoritative data from China as an example, in 2020, Chinese charitable organizations raised more than 8.2 billion yuan through 20 official Internet fundraising platforms (People.cn, 2021). Additionally, according to the 2021 annual report released by Shuidichou (see Footnote 1; a well-known private online donation platform in China), the platform has also raised more than 10 billion yuan for four consecutive years (CRN.cn, 2022). Not only is the rapid development, but the online donation platform based on social media also shows a new feature: the presence of peer donation information based on social platform data (Hou et al., 2021). For example, Shuidichou presents individual donors with peer donation information (e.g., number of donated peers) based on data obtained from social media platforms, which undoubtedly provides the possibility for the peer influence to play a role in the decision-making process of online donors.

Extant literature has intensively investigated the psychological mechanisms in donation decisions. For existing studies on determinants and antecedents of donation decision, emotions (e.g., empathy, guilt, and fear; Hibbert et al., 2007; Basil et al., 2008), warm glow (Gleasure and Feller, 2016), etc., were reported as potential motivations in making donation decision. In addition, the results of the latest research highlighted the critical role of perceived credibility (trust) and empathy in donors’ donation decisions (Liu et al., 2018; Chen et al., 2021), where Chen et al. (2021) further abstracted the donation decision as a dual-process approach (cognitive and emotional). However, these articles paid most of their attention to discovering discrete psychological factors originating from donors themselves. Although some of the existing research took organization-related factors (Fajardo et al., 2018) and social norms (DellaVigna et al., 2012; Erceg et al., 2018) into consideration, the discussion of their impact on donation decisions’ psychological mechanisms was missing. Few studies have examined the possible influence of external factors on the psychological process of donation decision-making.

As mentioned above, online donation platforms have started to demonstrate individual donors with peer information (e.g., number of donated peers). Peer influence means getting a balance between being oneself and conforming to group behavior (Hou et al., 2021), which indicates that the external influence of peers on the psychological process of donation decision should be considered in combination with individual factors. However, to the authors’ best knowledge, only two studies have investigated the impact of peer influence on online donation (Park and Shin, 2017; Hou et al., 2021), and both of them reported its positive effect on final donation decision without discussing their possible impact on decisions’ psychological mechanisms. Therefore, the current study aimed to address the question of *how peer influence (e.g., number of donated peers) affects the psychological process of donation decisions in the online donation context?* In detail *(1) Will it impact the emotional approach represented by empathy? (2) Will it impact the cognitive approach represented by trust? Or (3) Is there any new stage being introduced to donation decisions’ psychological process?*

Decision-making is a complicated process that includes various psychological and neural activities (Jin et al., 2017b). To better unveil the psychological process of peer-influenced online donation decisions and possibly link discrete psychological factors over time, the event-related potential (ERP) technic was employed. With its millisecond temporal resolution, ERP enables the assessment of the time course of brain responses underlying charitable donations (Carlson et al., 2015; Li et al., 2021). A considerable number of studies in Decision Neuroscience and, in particular, donation decisions, have shown its value in exploring the psychological and neural dynamics of donation decision processing (San Martin et al., 2016; Teng et al., 2018; Luo et al., 2019; Li et al., 2021). Thus, it provides foundations for us to conduct a study employing ERP to investigate the impact of the number of donated peers (in two levels: large and small) on the psychological process of donation decisions by recognizing donors’ neural mechanisms. Moreover, ERP contains various components (e.g., P2, N2, P3, and FRN), and each of them has its unique indications for the psychological process (San Martin et al., 2016; Wang et al., 2016; Teng et al., 2018; Luo et al., 2019; Li et al., 2021). Based on previous Decision Neuroscience studies, the current study examined three ERP components that are closely related to attention resources distribution (P2), decisional conflicts detection (N2), and reward anticipation and incentive formation (P3).

## Literature Review and Hypotheses Development

### Literature Review

As mentioned in the “Introduction”, peer influence was defined as getting a balance between being oneself and conforming to group behavior (Hou et al., 2021). In detail, it is a dyadic process by which an individual shapes him/her behavior, beliefs, or attitudes according to what the other individuals in the social system think, express, or how they behave (Leenders, 2002). The “being oneself” part of the definition means that the dual-process approach (Liu et al., 2018; Hou et al., 2021) of donation decisions caused by personal factors should be retained to a certain extent in the current peer-influenced context, which means, in the online donation decision that presents peer donation information, the decision-making process of empathy as emotional motivation and trust formation as cognitive precondition (Liu et al., 2018; Hou et al., 2021) may still exist.

But of more concern is the need for behavioral conformity in donors under peer influence, i.e., the “conforming to group behavior” part of the definition. Previous research has shown that, while the number of friends was increased, the increased social demand (from friends) could facilitate empathy (Wölfer et al., 2012; Morelli et al., 2017). Some studies later also suggest that social selection and socialization of peer influence will promote teens to select to be friends with people of similar features (Brechwald and Prinstein, 2011; Lewis et al., 2012), and being with friends with high empathy will further enhance their level of empathy (Miklikowska et al., 2022). These suggestions indicate that in the current study, a large number of donated peers will potentially promote the emotional process by facilitating the arousal of empathy. In addition, as the formation of trust is an essential cognitive precondition of donation decisions, the presentation of peer influence can further assist this process, both in reducing risk aversion (Ahern et al., 2014) and increasing perceived credibility (Ozdemir et al., 2020).

Furthermore, peer influence in online donations may prompt donors to pay more attention to the satisfaction of conformity needs and the positive emotional rewards that come with fulfilling the need. Conformity (Cialdini and Goldstein, 2004) clearly demonstrate the impact of the response of others on the actions of individuals. In detail, conformity provides an opportunity for individuals to consciously and deliberately gain social approval from others and build rewarding relationships while, in the process, increasing self-esteem (Cialdini and Goldstein, 2004). The satisfaction of relatedness need, one of the Basic Psychological Needs (Deci and Ryan, 2000), has been proved to arouse various positive emotions further (e.g., increasing sense of well-being; Dunn et al., 2014; Dunn and Norton, 2014; Zhang et al., 2021). In addition, some studies have put forward similar views from the perspective of social media characteristics. They believed that due to the non-anonymity characteristic of social media (Wallace et al., 2017), donors on social media might not be “pure altruism (Bénabou and Tirole, 2006)” and they might pursue a reward of self-satisfaction (e.g., self-presentation and seeking social recognition) through conspicuous donations on social media (Grace and Griffin, 2006, 2009). In general, peer-influenced donors are more likely to focus on the satisfaction of some of their own needs and be motivated by the rewards that come with the satisfaction. In other words, in the context of current research, the donors’ donation decision-making process is likely to include a stage of reward anticipation and the formation of corresponding emotional motivation.

### Behavioral Hypothesis

The positive effect of peer influence has been proven in previous studies (Messer et al., 2017; Park and Shin, 2017; Hou et al., 2021). In the current study, a large number of donated peers means a considerable number of peers on social media have made the donation decision, indicating a substantial peer influence. In contrast, the small number of donated peers means vice versa. Thus, we developed the behavioral hypotheses in conformity with previous studies:

*H1*: In peer-influenced online donation decisions, compared with the condition of small number of donated peers, the condition of large number of donated peers will result in donors’ higher donation willingness.

### ERP Hypothesis

According to the donation decision-making pattern proposed above, there seems to be a process of attention resource allocation between the altruism-oriented empathetic stimuli and potentially rewarding stimuli motivated by self-interest. Meanwhile, as different numbers of donated peers may reflect different levels of perceived credibility, there is likely to be a risk detection process. Additionally, as mentioned above, a process of reward anticipation and the formation of corresponding emotional motivation may exist. Therefore, in investigating the effect of peer influence on the psychological process of online donation decisions, the present study focused on three ERP components: attention resources distribution (P2), decisional risk detection (N2), and reward anticipation and incentive formation (P3).

#### P2 Hypothesis

P2 is a relatively early positive ERP component over frontal regions that presumably reflects the early assessment of stimuli (Polezzi et al., 2008) and occurs approximately 200 ms after the stimulus onset. It is an attention-related component towards emotion that indicates early rapid automatic activity, followed by the progressive recruitment of slow, elaborative, and semantic processing under voluntary control (Correll et al., 2006; Kanske et al., 2011; Ma et al., 2014; Jin et al., 2017b).

On the one hand, a considerable number of studies have indicated that negative or less positive stimuli will induce a greater P2 amplitude than positive ones (Carretié et al., 2001; Huang and Luo, 2006; Wang et al., 2012; Jin et al., 2017b). In the present study, as we mentioned in the “Literature Review” that increasing the number of peers will facilitate empathy (as the result of increased social demand; Wölfer et al., 2012; Morelli et al., 2017), the large number of donated peers may represent more positive emotional stimuli than the low number condition. Thus, if donors’ attention is primarily distributed to altruism-oriented empathetic stimuli, which means the empathy approach to donation decision dominates the early stage of neural activity, the low number of donated peers may elicit a larger P2 amplitude than the large number condition.

On the other hand, previous research shows that P2 is sensitive to the emotional evaluation of prospective rewards, which means high perceived rewards will elicit a higher P2 amplitude than low perceived rewards (Martin and Potts, 2004; Potts et al., 2006; Flores et al., 2015). In the current study, as we mentioned above, donors will be inclined to pay more attention to the fulfilment of their own needs when donating on social media (Wallace et al., 2017), especially the need for conformity when peer influence is present (Cialdini and Goldstein, 2004), and the satisfaction of those needs can provide positive emotional rewards (Cialdini and Goldstein, 2004; Dunn et al., 2014; Dunn and Norton, 2014; Zhang et al., 2021). We predict that the number of donated peers, which introduces peer influence in the social media donation, may further promote the motivation of donors to fulfil their own needs. Thus, donors may primarily distribute their attention to rewarding stimuli. In other words, the reward anticipation process is more likely to dominate the decision-making. Since a large number of donated peers indicates higher perceived rewards, we made a reasonable hypothesis:

*H2*: In peer-influenced online donation decisions, the condition of large number of donated peers may indicate higher perceived rewards and elicit a larger P2 amplitude than the condition of small number of donated peers.

#### N2 Hypothesis

N2 is another frequently studied negative component with a wave peaking at approximately 200–350 ms after stimulus onset (Folstein and Van Petten, 2008; Jin et al., 2017b). A considerable number of previous studies have reported that the amplitude of N2 is positively correlated with conflict in decision-making process (Ma et al., 2007; Folstein and Van Petten, 2008; Spapé et al., 2011; Jin et al., 2017b). More recent studies have suggested that N2 is also sensitive to decisional risk (Wang et al., 2016; Jin et al., 2017b) since the high perceived risk in the decision process can make the decision more difficult and lead to increase decisional conflict (Wang et al., 2016; Jin et al., 2017b).

As Chen et al. (2021) has discovered the trust-based cognitive approach of charitable donation decision, the role of trust may also exist in the current scenario. Meanwhile, since peer effect has been proved to reduce risk aversion (Ahern et al., 2014) and facilitate the formation of affective trust (Ozdemir et al., 2020), in the present study, compared with the small number of donated peers, the large number of donated peers illustrates a general recognition of the donation program by peers in the social media, which may positively promote the perceived trust that donors’ decisions depend on and reduce the effect of risk aversion, resulting in a lower level of decisional conflict. Thus, the following hypothesis was proposed.

*H3*: In peer-influenced online donation decisions, the condition of small number of donated peers may indicate a higher level of decisional conflict and elicit a larger N2 amplitude than the condition of large number of donated peers.

#### P3 Hypothesis

P3 is a positive-going component at centro-parietal recording sites that occurs approximately 300–600 ms post-stimulus onset (Euser et al., 2011; Yu et al., 2020). Although both the FRN and P3 are deemed the representative ERP components in reflecting reward anticipation, previous studies suggested that P3 is more stable in response to reward valence (Kujawa et al., 2013; Pfabigan et al., 2014; Zheng et al., 2015). Existing research suggested that P3 can reflect reward magnitude (e.g., large reward/small reward), which may indicate a conscious, top–down elaboration of the motivational significance of the outcome (Wu and Zhou, 2009; Leng and Zhou, 2010; Li et al., 2010; Novak and Foti, 2015). Notably, P3 may reflect affective processes by signaling the motivational salience of reward feedback (San Martín, 2012). More recent studies also found the sensitivity of P3 amplitude in detecting positive social feedback among healthy individuals, indicating an encoding bias for desirable and self-affirming information (Van der Veen et al., 2016; Funkhouser et al., 2020; Yao et al., 2021).

As mentioned above, the conformity need embodied in peer influence may bring donors an opportunity to consciously gain self-approval and build rewarding relationships (Cialdini and Goldstein, 2004). The satisfaction of relatedness needs can further arouse positive emotional rewards, like increasing the sense of well-being (Dunn et al., 2014; Dunn and Norton, 2014; Zhang et al., 2021). In the current study, since peer-influenced donors may be more inclined to satisfy their own needs and gain the potential emotional rewards, especially in the social media context (Wallace et al., 2017), Compared with the small number of donated peers, the large number condition may signal higher demand for conformity and relatedness needs and a higher degree of anticipated emotional rewards, indicating more significant donation motivations. Therefore, we made the following hypothesis:

*H4*: In peer-influenced online donation decisions, the condition of large number of donated peers may lead to a stronger motivation to make the donation decision and elicit a larger P3 amplitude than the condition of small number of donated peers.

## Materials and Methods

### Participants

To determine how many subjects are needed, we check *via* a power analysis using G^*^Power 3.1 (Faul et al., 2009). Set an effect size of 0.4, a power of 0.8, and an alpha level of 0.05, the result of the power analysis estimated a sample size of 27. Thus, 30 college students (17 males, 18–26 years old, *M* = 19.740, SD = 1.853) from Ningbo University were randomly recruited as subjects. All of them were native Chinese speakers with normal or corrected-to-normal vision. They were self-reported right-handed and had no history of neurological disorders and mental diseases. The participants signed written informed consent before the experiment and were paid RMB 40 (around $6) as remuneration. One of the participants was excluded due to excessive artifacts in the electroencephalogram (EEG) recordings. Ultimately, the number used for data analysis was 29. The study was approved by the Internal Review Board of the Academy of Neuroeconomics and Neuromanagement at Ningbo University.

### Stimuli

**Figure 1:**
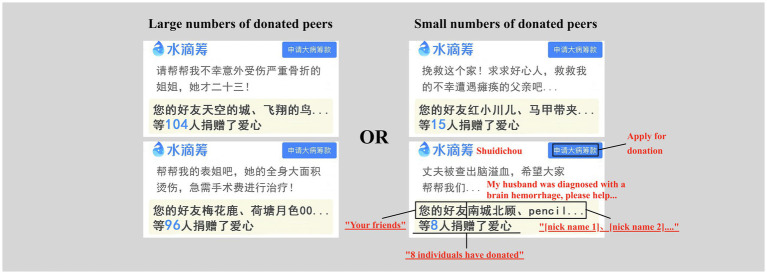
Demonstration of stimulus photos in the two conditions (the large vs. small number of donated peers). Four schematic diagrams were showed, with two of them corresponding to the condition of large number of donated peers (left two photos) and two of them corresponding to the condition of small number of donated peers (right two photos).

The entire experiment contained 80 stimuli. It had a total of 20 stimulus pictures (800 × 394 pixels) of two types (large number of donated peers vs. small number of donated peers), with ten pictures for each type. All the stimulus pictures used in the formal experiment were prepared by referring to the page layout of Shuidichou (see Footnote 1) with the information of donation objects’ requirement in the center and the bolded number of donated peers below (see Figure 1). To minimize the impact of irrelevant information, we controlled the size, color, and font of words to be consistent and manipulated the word count of fundraising slogans and page backgrounds. Ninety-five to 105 donated peers (without 100) and 5–15 donated peers (without 10) were set as large and small numbers, respectively.

### Procedure

The subjects were asked to enter a soundproof room and sit on a comfortable chair in the experiment. The chair was 100 cm away from a computer-controlled monitor (1,280 × 1,024 pixels) with a refresh rate of 60 Hz. Before the formal experiment started, each participant received an instruction paper detailing the current experiment’s task, procedure, and announcements. They were explicitly told that they should imagine that they currently are browsing some ongoing donation programs in a social media-based online donation platform and are going to determine their willingness to donate, and then they should respond by fulling the on-screen Likert scale through the keyboard provided. Stimuli presentation and data collection were controlled by E-Prime 3.0 software (PST, Psychology Software Tools, Inc.).

**Figure 2:**
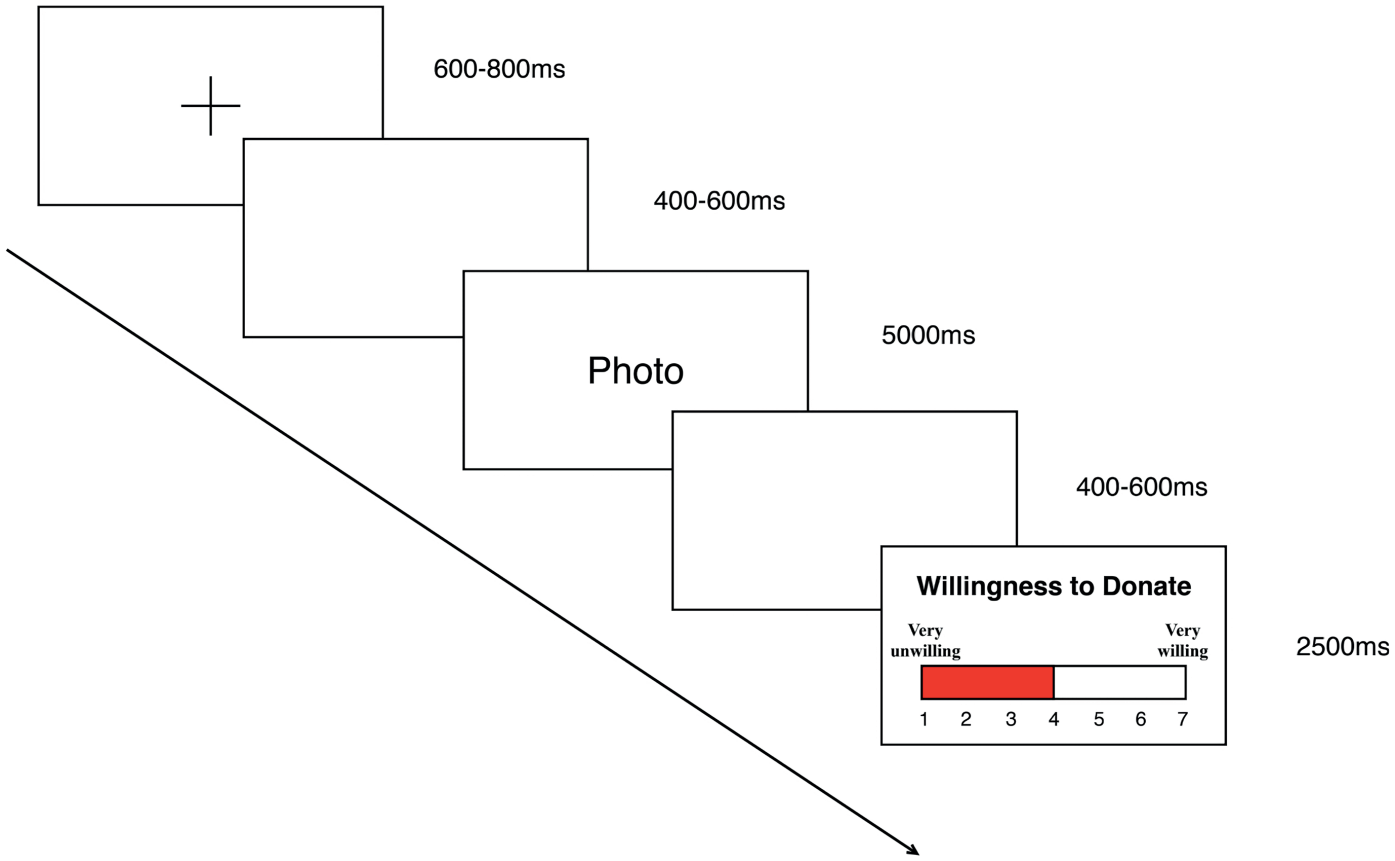
Single trial of the experimental procedure. Participants are instructed to report their donation intention under the condition that the number of donated peers was large or small. EEGs and behavioral data were recorded throughout the experiment.

As shown in Figure 2, each trial began with a black cross against a white background for 600–800 ms, followed by a 400–600 ms blank screen. Then, a target stimulus of an ongoing donation program and the number of donated peers was shown for 5,000 ms. After another 400–600 ms blank screen, subjects could use the keypad provided to report their “willingness to donate” through an on-screen Likert scale ranging from 1 (very unwilling) to 7 (very willing) in 2,500 ms. In the formal experiment, each stimulus picture will be repeated four times (80 in total with 40 in the large number of donated peers and 40 in the small number of donated peers). All 80 stimulus pictures are randomly assigned to four blocks (20 trials each). And all trials were presented randomly in the experiment.

Especially, the following details about the experiment need to be explained: (1) during the interval of each block, subjects could take a short break and the whole formal experiment lasts for around 12 min; and (2) subjects were told before the experiment that the donating decision for each donation program should be made independently.

### Behavioral Data Recording and Analysis

In this experiment, Behavioral data of donation intention was the dependent variable and was automatically collected by E-Prime 3.0 Software. A pairwise *t*-test was performed to examine the discrepancy between the average donation intention of the large number of donated peers and the small number of donated peers.

### EEG Recording and Analysis

A 64 Ag/AgCl electrodes cap and a Neuroscan Synamp2 Amplifier (Curry8, Neurosoft Labs, Inc.) were used to record EEG data at a sample rate of 500 Hz. The EEG signal was recorded at the decision screen (determining the “willingness to donate”) of each trail. The left mastoid was used for reference, and a cephalic location between PFz and Fz was used as the ground. Data were off-line transferred to the average of the left and right mastoid references. The electrooculogram (EOG) was recorded from electrodes placed at 10 mm from the lateral canthi of both eyes (horizontal EOG) and above and below the left eye (vertical EOG). EOG artifacts were off-line corrected for all subjects, following the method provided by Semlitsch et al. (1986). The electrode impedances were controlled below 5 kΩ during the whole experiment. EEG data preprocessing was performed using the EEGLAB toolbox (Delorme and Makeig, 2004) and MATLAB (R2013a, The MathWorks, Inc., Natick, MA, United States). First, EEG data were re-referenced to the left and right mastoids average, bandpass filtered to a range of 0.1–30 Hz, epoched from − 200 to + 800 ms surrounding the simulation screen onset and took the baseline activity from – 200 to 0 ms preceding the target. Independent component analysis was computed using the EEGLAB toolbox. And then, ICs representing eye blinks or other artifacts were removed from the EEG data. Finally, the EEG recordings over each recording site for each participant were averaged within two conditions (large number of donated peers vs. small number of donated peers), respectively.

As mentioned in the “Introduction,” three ERP components were analyzed in the current experience, namely P2, N2, and P3. Based on visual observation and the guideline proposed by Picton et al. (2000), the following representative channels and time windows were selected: (1) for P2, nine electrodes in the frontal-central area (F3, Fz, F4, FC3, FCz, FC4, C3, Cz, and C4; Jin et al., 2017b) were selected in the time window from 230 to 280 ms; (2) for N2, nine electrodes in the frontal–central area (F3, Fz, F4, FC3, FCz, FC4, C3, Cz, and C4; Jin et al., 2017a) were selected in the time window from 280 to 330 ms; (3) for P3, nine electrodes in the whole brain area (C3, Cz, C4, CP3, CPz, CP4, P3, Pz, and P4; Cai et al., 2021) were selected in the time window from 330 to 380 ms. The mean amplitudes of P2, N2, and P3 in each condition were extracted separately according to the time window, and two-way repeated ANOVA analyses were conducted for each condition with the two within-subject factors (large/small number of donated peers and electrodes). Greenhouse–Geisser corrections were used for determining significances (Greenhouse and Geisser, 1959) and partial eta squared values ( $\eta_{p}^{2}$ ) were reported to demonstrate the size of effects in ANOVA models where 0.05 represents a small effect, 0.1 represents a medium effect, and 0.2 represents a large effect (Muller, 1989).

## Results

### Behavioral Results

**Figure 3:**
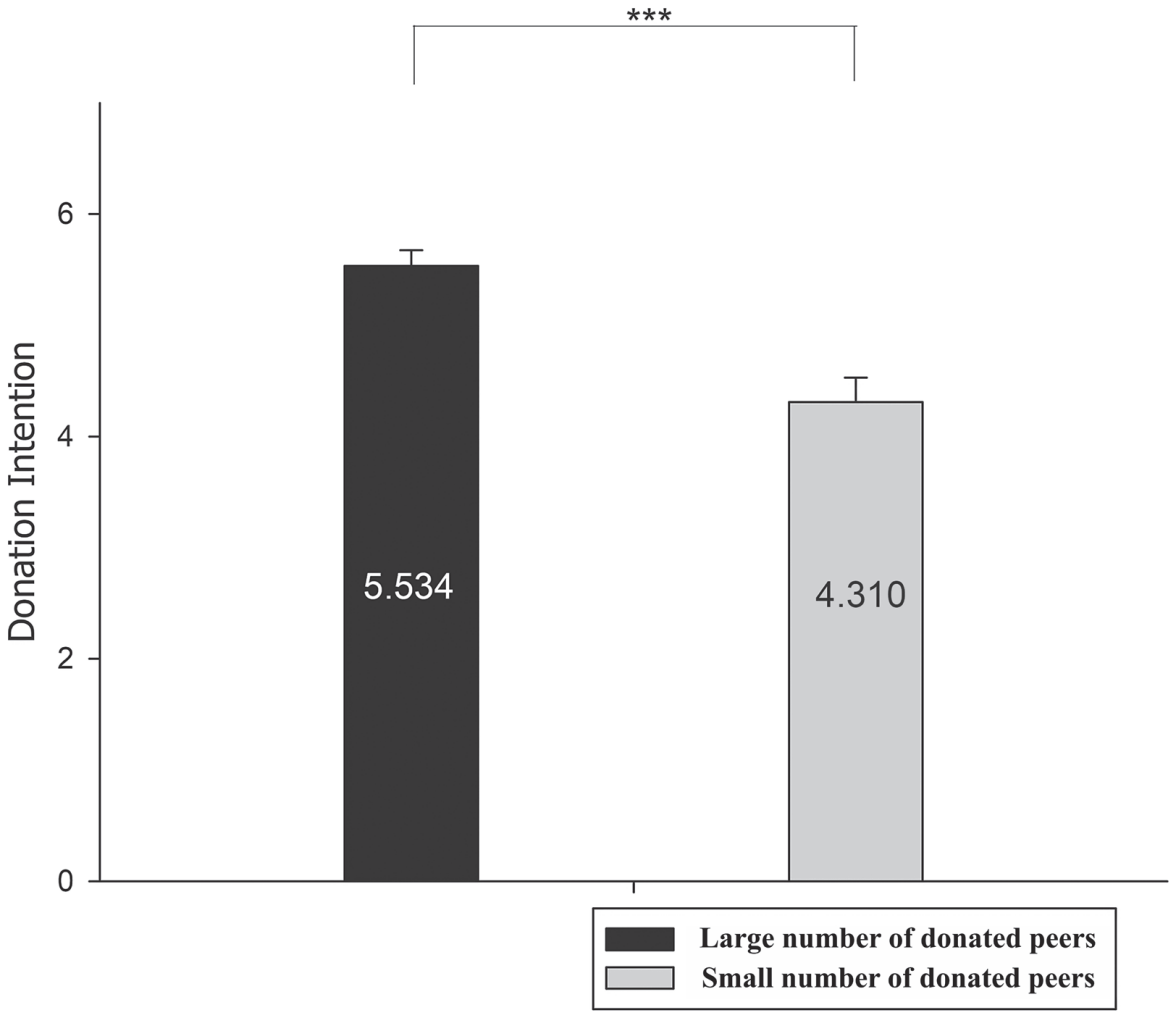
The behavioral results of donation intention. The black bar represents the average donation intention in the large number of donated peers, whereas the grey bar represents the average donation intention in the small number of donated peers. ^***^p < 0.001.

Behavioral results are shown in Figure 3. A pairwise *t*-test was performed for the donation intention between the different numbers of donated peers. The results showed a significant main effect, *t*(1,28) = 4.102, *p* < 0.001, which indicate that the donation intention in the situation of large number of donated peers (*M* = 5.534, SE = 0.141) is significantly higher than the situation of large number of donated peers (*M* = 4.310, SE = 0.220).

### ERP Results

#### P2

**Figure 4:**
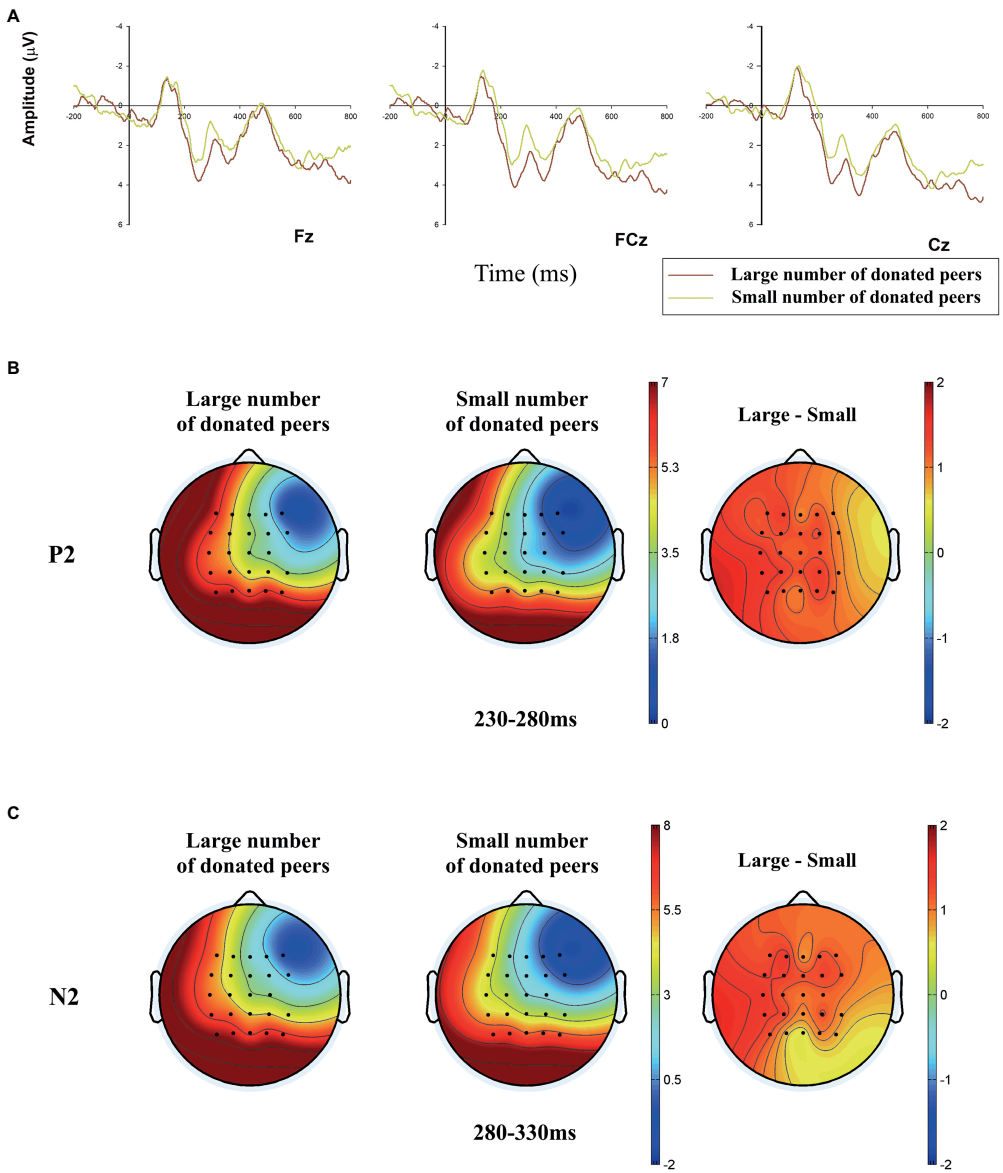
Grand-averaged event-related potential (ERP) waveforms of P2 and N2 in the three electrodes of frontal-to-central brain region, and related brain topographies. **(A)** The demonstration of P2 and N2 amplitudes in the two conditions (large vs. small number of donated peers) in representative electrodes (Fz, FCz, and Cz); **(B)** The P2 brain topographies of the two conditions in the time window of 230–280 ms; **(C)** The N2 brain topographies of the two conditions in the time window of 280–330 ms.

The 2 (large number of donated peers vs. small number of donated peers) × 9 (electrodes: F3, Fz, F4, FC3, FCz, FC4, C3, Cz, and C4) two-way repeated-measures ANOVA analysis for the mean amplitudes of P2 (positive polarity: larger voltage value means larger amplitude) was conducted in the time window of 230–280 ms (see Figures 4A,B). The main effect of the number of donated peers was observed, *F*(1,28) = 8.010, *p* = 0.009, $\eta_{p}^{2}$ = 0.222, indicating that the average P2 amplitudes of subjects who encountered large number of donated peers (*M* = 3.776 μV, SE = 0.613 μV) is significantly larger than who faced small number of donated peers (*M* = 2.651 μV, SE = 0.531 μV).

#### N2

The results of two-way 2 (large number of donated peers vs. small number of donated peers) × 9 (electrodes: F3, Fz, F4, FC3, FCz, FC4, C3, Cz, and C4) repeated measures ANOVA analysis for N2 in 280–330 ms were shown in Figures 4A,C, which suggested that the condition of large number of donated peers (*M* = 3.009 μV, SE = 0.603 μV) elicited a significantly smaller amplitudes compared to the small number of donated peers condition (*M* = 1.832 μV, SE = 0.603 μV), *F*(1,28) = 10.248, *p* = 0.003, $\eta_{p}^{2}$ = 0.268. Since N2 is a negative polarity ERP component, a smaller voltage value means a larger amplitude.

#### P3

**Figure 5:**
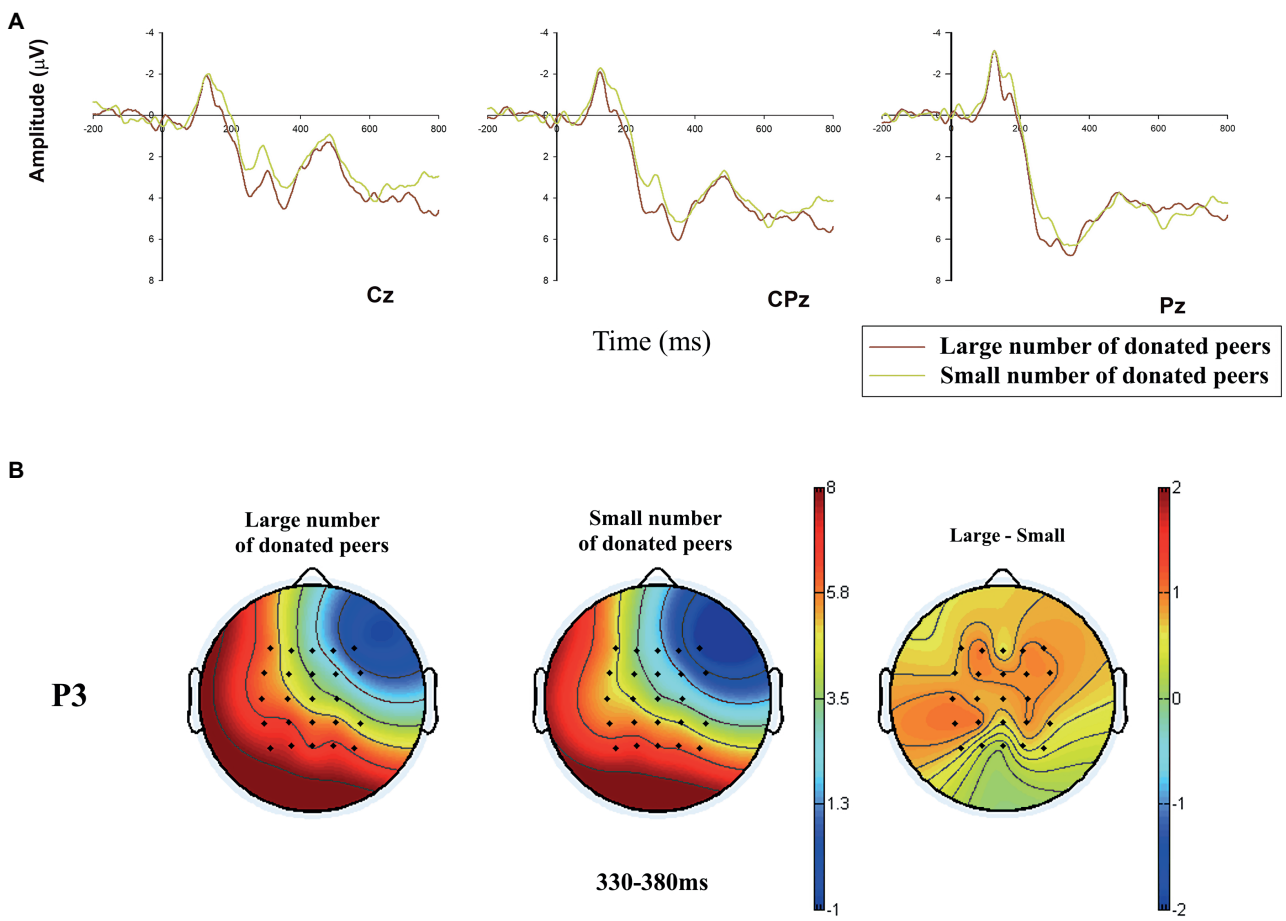
Grand-averaged event-related potential (ERP) waveforms of P3 in three electrodes of central-to-parietal brain region, and related brain topographies. **(A)** Demonstration of P3 amplitudes in the two conditions (large vs. small number of donated peers) in representative electrodes (Cz, CPz, and Pz); **(B)** The P3 brain topographies of the two conditions at the time window of 330–380 ms.

The two-way 2 (large number of donated peers vs. small number of donated peers) × 9 (electrodes: C3, Cz, C4, CP3, CPz, CP4, P3, Pz, and P4) repeated measures ANOVA analysis for the mean amplitudes of P3 (positive polarity: larger voltage value means larger amplitude) in the time window of 330–380 ms indicated a significant main effect for the two conditions, *F*(1,28) = 5.795, *p* = 0.023, $\eta_{p}^{2}$ = 0.171. The large number of donated peers (*M* = 5.504 μV, SE = 0.626 μV) elicited a larger P3 mean amplitude than the small number of donated peers (*M* = 4.778 μV, SE = 0.579 μV) as shown in Figure 5.

## Discussion

Previous studies have successfully investigated various discrete, individual donor-oriented psychological factors in the donation decision-making process. However, few studies have examined the possible influence of external factors on the psychological process of donation decision-making. As peer information (e.g., the number of donated peers) has been presented in some online donation platform (e.g., Shuidichou), the potential effect of peer influence, as an external factor, on the psychological decision-making process should be examined. Therefore, current study used the number of donated peers to explore the neural and psychological process of peer-influenced online donation decision with the assistance of ERP, hoping to gain some new insights. Thirty college students were recruited, and a lab experiment was conducted. The behavioral result showed that the donation willingness in large number condition was significantly higher than that in the small number condition. Meanwhile, the ERP results showed that larger P2 amplitudes, smaller N2 amplitudes, and larger P3 amplitudes were elicited in the condition when a large rather than a small number of donated peers was presented.

The behavioral results of this study once again confirmed the findings of previous studies: peer influence can have a significant impact on donors’ donation behaviors (Messer et al., 2017; Park and Shin, 2017; Hou et al., 2021). More specifically, in the experimental condition simulating donation decision in a social media-based online donation platform, donors’ donation decisions were influenced by the presented numbers of donated peers. The large number of donated peers resulted in a high willingness to donate, and the small number of donated peers was the opposite. Previous studies have suggested how the donors reached the result (Messer et al., 2017; Park and Shin, 2017). With the support of ERP results, this study discussed the underlying psychological process of the result in detail below.

The ERP results showed that smaller amplitudes of P2 were elicited in the condition of a small number of donated peers compared with a large number of donated peers. Thus, H2 was supported. As mentioned above, existing studies have found that the P2 component is a symbol of emotional evaluation of prospective rewards, and a higher perceived reward will elicit a larger P2 amplitude (Martin and Potts, 2004; Potts et al., 2006; Flores et al., 2015). This result suggests that, in the current context, although past research considered emotional processing represented by empathy exists in donation decision making (Chen et al., 2021). However, due to the dual effects of the characteristics of social media (Wallace et al., 2017) and peer influence, donors showed higher attention to emotional reward perception in the early neural activity of decision-making, which is consistent with the previous discussion in the “Hypothesis”. Based on this, since a large number of donated peers represented more attention from peers on the donation project, compared with the low number condition-based decisions, decisions based on large number stimuli would bring a higher level of satisfaction of conformity and relatedness needs, that is, these decisions indicated a higher level of perspective emotional rewards (Dunn et al., 2014; Dunn and Norton, 2014; Zhang et al., 2021). In addition, some other studies may also provide some support for our conclusion. First, previous research shows that reward perception can be further decomposed into a cue-evaluation stage that a cue is presented and provides the information on the possible outcomes of the current trial (which can be represented by P2 amplitudes), and a feedback anticipation stage (Broyd et al., 2012; Novak and Foti, 2015; Pornpattananangkul and Nusslock, 2015; Glazer et al., 2018). Therefore, it is possible that the perception of the prospective reward outweighs the attention to the emotional stimulus itself in the P2 stage. Second, peer influence, along with the effect of conformity, was considered to be more pronounced in collectivist countries (e.g., East Asian countries) than in individualist countries (Markus and Kitayama, 1991), which indicates that the social context of the present study may further promote the role of peer influence. In conclusion, the large number condition evoked higher P2 amplitudes suggesting that a reward perception process based on rapid automatic analysis of stimuli was involved in the early psychological process of peer-influenced donation decisions.

During the time window of N2 components, the analysis showed the amplitudes of N2 were larger in the condition of a small number of donated peers than in the large number condition. Thus, H3 is supported. According to previous studies, N2 is sensitive to decisional conflicts (Ma et al., 2007; Folstein and Van Petten, 2008; Jin et al., 2017b), and higher perceived risk during decision-making will further lead to increased decisional conflict (Wang et al., 2016; Jin et al., 2017b). Since previous studies have consistently emphasized the importance of trust forming (or a high level of perceived credibility) in making the final donation decision (Liu et al., 2018; Chen et al., 2021), the trust-based cognitive approach to charitable donation may still exist in peer-influenced online donation decisions. Moreover, peer influence has been shown to further promote trust formation (Ozdemir et al., 2020) and reduce risk aversion (Ahern et al., 2014). In the present context, the information of the large number of donated peers illustrated a general recognition of the donation program among the majority of peers and thus, promoted the perceived credibility of the donation while reducing the decisional risk. Compared with the large number condition, the small number of donated peers indicated a higher potential risk of the donation, and a larger N2 amplitude was elicited. Therefore, the result of N2 supported that a conflict and risk detection procedure existed in the peer-influenced donation decision-making process.

The P3 component was found following the N2 component in this study. The evaluation of P3 component indicated that the large number of donated peers evoked a larger P3 amplitude than the small number condition. Thus, H4 is supported. Existing literature has continuously demonstrated that a large reward can elicit a larger P3 amplitude than a small reward when evaluating the motivational significance of stimuli (Nieuwenhuis et al., 2005; Schevernels et al., 2014; Wu et al., 2016; Zhang et al., 2017) and P3 may reflect affective processes by signaling the motivational salience of reward feedback (San Martín, 2012). Meanwhile, P3 has also been found to be sensitive to positive social feedback (Van der Veen et al., 2016; Funkhouser et al., 2020; Yao et al., 2021). The results suggested that, in the current study, donors tended to use social donation to meet some of their own needs (e.g., conformity and relatedness needs) and gain emotional rewards accordingly since the conformity can bring self-approvement and rewarding relationships (Cialdini and Goldstein, 2004), and the satisfaction of relatedness need can further arouse various positive emotions (e.g., the sense of well-being; Dunn et al., 2014; Dunn and Norton, 2014; Zhang et al., 2021). Compared with the large number condition, the small number of donated peers indicated a lower magnitude of emotional reward and thus, indicating a lower motivation for the donors to form their final donation decision (reflected by a smaller amplitude of P3). The result of P3 supported that a reward anticipation and motivation formation procedure existed in the psychological process of peer-influenced online donation decisions.

Based on the above results and what we have discussed, this study has the following implications. Theoretically, we successfully replicated the effect of peer influence in the online donation scenarios and once again proved the impact of peer influence on donation behavior, which was consistent with previous studies (Messer et al., 2017; Park and Shin, 2017; Hou et al., 2021). In addition, we also suggested new insights into the psychological process of peer-influenced donation decisions from the perspective of ERP. First, the process that perceived expected rewards dominated the emotional approach in peer-influenced donation decisions. The need for conformity and relatedness brought about by peers and the prospection to obtain emotional rewards after the need was satisfied motivated donors to allocate more attention resources to high prospective reward stimuli (large number of donated peers) in the early decision-making stage (represented by a larger amplitude of P2 in the large number condition). Second, the cognitive approach represented by trust formation still existed in the decision-making process, where strong peer influence represented by the large number of donated peers promoted decisional risk reduction and trust formation (represented by a small amplitude of N2 in the large number condition). Third, in addition to the above-mentioned early neural activities, the P3 component suggested that a process of elaborate processing of perceived reward outcomes and the formation of corresponding motivations existed in peer-influenced donation decisions (represented by a large amplitude of P3 in the large number condition). As an extension of the intentional evaluation of reward stimuli in the previous P2 component, the P3 stage allowed donors to anticipate the reward outcomes (Broyd et al., 2012; Novak and Foti, 2015; Pornpattananangkul and Nusslock, 2015; Glazer et al., 2018), and the large number of donated peers corresponded to high salience motivation, which guided the formation of final decisions.

Charities have become one of the essential ways to help marginalized and vulnerable social groups (Erceg et al., 2018), and online donation is also gradually becoming an important part of charitable giving. Therefore, practically, the current study’s demonstration of peer influence in promoting donation decisions and the revealing of the underlying early psychological process of the decision suggested that, on the premise of protecting user privacy, online donation platforms can make full use of the advantages of the network to increase the influence of peers in donation projects, which will help to raise funds better.

We cannot deny that there are some limitations to the current study. First, due to limited research resources, other information shown on the donation page of the social media-based donation platform is not investigated, for example, the donation amount of peers. A study has indicated that donors have a potential computation process when making donation decisions (Messer et al., 2017). Thus, this leads to an open question of what the possible process of donation decision is when the donation amount of peers present and whether there are potential interactions between the number of donated peers and the amount of their donation or not. Secondly, although we tried to simulate the actual scenario in the strict ERP experimental environment, discrepancies still existed between the lab environment and actual donation conditions. Therefore, it should be careful when generalizing the current study’s findings to the real world. Third, all we considered in the present study was large and small number of donated peers without setting a control group of large and small number of donors (not donated peers). Thus, the paradigm of the current study should be improved, and further ERP experiments should be conducted to replicate the present results. Moreover, the volunteers of this study were mainly college students. Participants with more diverse backgrounds should be recruited to form a more comprehensive view of general brain activities during donors’ donation decisions with peer influence present.

## Conclusion

This study aimed to explore the primary effect of peer influence on the donors’ psychological process when they make donation decisions on social media-based platforms with the assistance of event-related potential. Using large and small numbers of donated peers to represent different magnitudes of peer influence, the behavioral results stayed in conformity with previous studies, which suggested that peer influence positively affects the donors’ donation willingness. The ERP results further explained the behavioral results of the donation decision, indicating that three emotional or cognitive stages might exist. Chronologically, the donor first experienced early processing of prospective rewards (reflected by P2); and then a cognitive process of decisional risk detection and trust formation appeared (reflected by N2); in the later stage, based on the previous perceived emotional rewards, further elaborate evaluation of reward stimuli presented and the motivation formed accordingly, leading to final donation decisions (reflected by P3). The results of ERP strongly suggested that an emotional approach represented by reward anticipation and a cognitive approach of risk detection and trust formation exist in peer-influenced donation decisions. Based on these findings, the current study can help the online donation platforms understand their users’ psychological process of donation decisions and utilize the peer influence for better fundraising.

## Data Availability Statement

The raw data supporting the conclusions of this article will be made available by the authors, without undue reservation.

## Ethics Statement

The studies involving human participants were reviewed and approved by the Internal Review Board of the Academy of Neuroeconomics and Neuromanagement at Ningbo University. The patients/participants provided their written informed consent to participate in this study.

## Author Contributions

YY made substantial contributions and participated in all aspects of the paper, conducted the experiment, analyzed the data, and wrote the manuscript. PJ made substantial contributions to the work and participated in all aspects of the paper. WZ oversaw the study and managed every part of the research. All authors read and approved the final manuscript. All authors contributed to the article and approved the submitted version.

## Funding

This work was supported by the Project of Philosophy and Social Science Key Research Base of Zhejiang Province (grant number 20JDZD024); Zhejiang Provincial Natural Science Foundation of China (grant number LQ20G020010); National Nature Science Foundation of China (grant number 71942002); and the Fundamental Research Funds for the Provincial Universities of Zhejiang (grant number SJWY2020001). The funders had no role in the study design, collection, data analysis, or interpretation of the data, in the writing of the report, or in the decision to submit the article for publication.

## Conflict of Interest

The authors declare that the research was conducted in the absence of any commercial or financial relationships that could be construed as a potential conflict of interest.

## Publisher’s Note

All claims expressed in this article are solely those of the authors and do not necessarily represent those of their affiliated organizations, or those of the publisher, the editors and the reviewers. Any product that may be evaluated in this article, or claim that may be made by its manufacturer, is not guaranteed or endorsed by the publisher.
